# Supplementary material for: DNA origami presenting the receptor binding domain of SARS-CoV-2 elicit robust protective immune response
Source: Commun Biol. 2023 Mar 23;6:308. doi: 10.1038/s42003-023-04689-2 (PMC10034259; doi:10.1038/s42003-023-04689-2)
Supplement: Supplementary file 2 — Supplementary Information [file 42003_2023_4689_MOESM2_ESM.pdf]

Supporting Information

DNA Origami Presenting the Receptor Binding Domain of SARS-CoV-2 Elicit Robust Protective Immune Response

Esra Oktay[1], Farhang Alem[2], Keziah Hernandez[2], Michael Girgis[1], Christopher Green[3], Divita Mathur[4], Igor L. Medintz[3], Aarthi Narayanan[2], and Remi Veneziano[1*]*

[1] Department of Bioengineering, George Mason University, Fairfax, VA 22030, USA

[2] National Center for Biodefense and Infectious Diseases, George Mason University, Manassas, VA 20110, USA

[3]Center for Bio/Molecular Science and Engineering Code 6900, U.S. Naval Research Laboratory, Washington DC, USA;

[4]Department of Chemistry, Case Western Reserve University, Cleveland OH, USA

*Correspondence: rvenezia@gmu.edu and anaraya1@gmu.edu.

SUPPLEMENTARY TABLES

Supplementary Table 1 ssDNA scaffold sequence. The primer binding sequences are shown in red.

**PB ssDNA scaffold sequence**
**GCGACGATTTACAGAAGCAAGGTTATTCACTCACATATATTGATTTATGTACTGTTTCCATTAA<br>AAAAGGTAATTCAAATGAAATTGTTAAATGTAATTAATTTTGTTTTCTTGATGTTTGTTTCATC<br>ATCTTCTTTTGCTCAGGTAATTGAAATGAATAATTCGCCTCTGCGCGATTTTGTAACTTGGTAT<br>TCAAAGCAATCAGGCGAATCCGTTATTGTTTCTCCCGATGTAAAAGGTACTGTTACTGTATAT<br>TCATCTGACGTTAAACCTGAAAATCTACGCAATTTCTTTATTTCTGTTTTACGTGCTAATAATT<br>TTGATATGGTTGGTTCAATTCCTTCCATAATTCAGAAGTATAATCCAAACAATCAGGATTATAT<br>TGATGAATTGCCATCATCTGATAATCAGGAATATGATGATAATTCCGCTCCTTCTGGTGGTTT<br>CTTTGTTCCGCAAAATGATAATGTTACTCAAACTTTTAAAATTAATAACGTTCGGGCAAAGGAT<br>TTAATACGAGTTGTCGAATTGTTTGTAAAGTCTAATACTTCTAAATCCTCAAATGTATTATCTA<br>TTGACGGCTCTAATCTATTAGTTGTTAGTGCACCTAAAGATATTTTAGATAACCTTCCTCAATT<br>CCTTTCTACTGTTGATTTGCCAACTGACCAGATATTGATTGAGGGTTTGATATTTGAGGTTCA<br>GCAAGGTGATGCTTTAGATTTTTCATTTGCTGCTGGCTCTCAGCGTGGCACTGTTGCAGGCGG<br>TGTTAATACTGACCGCCTCACCTCTGTTTTATCTTCTGCTGGTGGTTCGTTCGGTATTTTTAAT<br>GGCGATGTTTTAGGGCTATCAGTTCGCGCATTAAAGACTAATAGCCATTCAAAAATATTGTCT<br>GTGCCACGTATTCTTACGCTTTCAGGTCAGAAGGGTTCTATCTCTGTTGGCCAGAATGTCCCT<br>TTTATTACTGGTCGTGTGACTGGTGAATCTGCCAATGTAAATAATCCATTTCAGACGATTGAG<br>CGTCAAAATGTAGGTATTTCCATGAGCGTTTTTCCTGTTGCAATGGCTGGCGGTAATATTGTT<br>CTGGATATTACCAGCAAGGCCGATAGTTTGAGTTCTTCTACTCAGGCAAGTGATGTTATTACT<br>AATCAAAGAAGTATTGCTACAACGGTTAATTTGCGTGATGGACAGACTCTTTTACTCGGTGGC<br>CTCACTGATTATAAAAACACTTCTCAAGATTCTGGCGTACCGTTCCTGTCTAAAATCCCTTTAA<br>TCGGCCTCCTGTTTAGCTCCCGCTCTGATTCCAACGAGGAAAGCACGTTATACGTGCTCGTCA<br>AAGCAACCATAGTACGCGCCCTGTAGCGGCGCATTAAGCGCGGCGGGTGTGGTGGTTACGCG<br>CAGCGTGACCGCTACACTTGCCAGCGCCCTAGCGCCCGCTCCTTTCGCTTTCTTCCCTTCCTT<br>TCTCGCCACGTTCGCCGGCTTTCCCCGTCAAGCTCTAAATCGGGGGCTCCCTTTAGGGTTCCG<br>ATTTAGTGCTTTACGGCACCTCGACCCCAAAAAACTTGATTTGGGTGATGGTTCACGTAGTGG<br>GCCATCGCCCTGATAGACGGTTTTTCGCCCTT**

Supplementary Table 2 Primer set for the PB ssDNA scaffold synthesis.

**PB forward primer**	**GCGACGATTTACAGAAGCAA**
**PB reverse primer**	AAGGGCGAAAAACCGTCTAT

Supplementary Table 3 Unmodified staple sequences for folding the PB.

**Name**	**Sequence**

**PB-2**	CCACCGAGTAATTTTTAAGAGTCTGTTCTTTGATTAGTTTTTTAATAACATC
**PB-3**	GGCCTTGCTGGTTTTTTAATATCCAGTAGACAGGAACTTTTTGGTACGCCAG
**PB-4**	GAAGGTTATCTTTTTTAAAATATCTTCGTCAATAGATTTTTTAATACATTTG
**PB-5**	ATATAATCGTTGGCAAATCAACAGTAGAAAG
**PB-6**	GAATTGAGTATCAGATGATGGCAATTCATCA
**PB-7**	CGCTGGCAAAGCGAAAGGAGCGGGTATTAAT
**PB-8**	TTTAAAAGCCTTTGCCCGAACGTCGCTAGGG
**PB-9**	GCAATACTCCATCACGCAAATTAATAGACTT
**PB-10**	TACAAACAAGGATTTAGAAGTATCCGTTGTA
**PB-11**	ATTCGACAACTTTTTTCGTATTAAATTTTGAGTAACATTTTTTTATCATTTTAATTATCATCATTTTTATTCCTGAT
**PB-12**	AGGAGCGGGCGGAACAAAGAAACATGATGAA
**PB-13**	ACAAACATACCTGAGCAAAAGAAGCACCAGA
**PB-14**	ATTAGAGCTAGGTGCACTAACAATAAGAATA
**PB-15**	CGTGGCACTTCTGACCTGAAAGCGCTAATAG
**PB-16**	GAGAGCCAAACAGAAATAAAGAAATTGCGTA
**PB-17**	GATTTTCAACCGCCTGCAACAGTGCCACGCT
**PB-18**	GGGAGAAACAATATATGTGAGTGGGCCCACT
**PB-19**	ACGTGAACACAGTAACAGTACCTTTTACATC
**PB-20**	GGTTTAACGTCTTTTTAGATGAATATCATCACCCAAATTTTTTCAAGTTTTT
**PB-21**	TCCTCGTTGGATTTTTATCAGAGCGGAAAACGCTCATTTTTTGGAAATACCTGGTGAGGCGGTTTTTTCAGTATTAAC
**PB-22**	ACGTGCTTTGGGGTCGAGGTGCCGTAAAGCA
**PB-23**	CTAAATCGGGTTGCTTTGACGAGCACGTATA
**PB-24**	AAAACAGAACATTTTGACGCTCAATCGTCTG
**PB-25**	AAATGGATCGAACGAACCACCAGCAGAAGAT
**PB-26**	TGGCGAGAAAGTTTTTGAAGGGAAGAAGTGTAGCGGTTTTTTCACGCTGCGC
**PB-27**	CAGTGAGGAATCTTGAGAAGTGTTGCCGCGC
**PB-28**	CCGCTACAGGGTTTTTCGCGTACTATGAACCCTAAAGTTTTTGGAGCCCCCG

**PB-29**	TTAATGCGGTAACCACCACACCCTTTATAAT
**PB-30**	CCCTCAATCAATTTTTTATCTGGTCACTGATTGTTTGTTTTTGATTATACTT
**PB-31**	ATCAAAATTATTTTTTTAGCACGTAAGCAGCAAATGATTTTTAAAATCTAAA
**PB-32**	CGCGCAGAGCTTTGAATACCAAGTAATTGAA
**PB-33**	CCAACCATCTGAATTATGGAAGGTACAAAAT
**PB-34**	TAGCCCTAATTAGTCTTTAATGCGACCTCAA
**PB-35**	ATATCAAAGCATCACCTTGCTGACGAACTGA
**PB-36**	TATTTACATTGTTTTTGCAGATTCACTGGCCAACAGATTTTTGATAGAACCC
**PB-37**	AGACAATATTTTTTTTTTGAATGGCTAAACATCGCCATTTTTTTAAAAATAC
**PB-38**	AAACTATCACTTGCCTGAGTAGAAATAAAAG
**PB-39**	GGACATTCCAGTCACACGACCAGTAGAACTC
**PB-40**	GGCGAATTATTTTTTTCATTTCAATTCAAGAAAACAATTTTTAATTAATTAC
**PB-41**	TAATGGAAACATTTTTGTACATAAATCAATAACGGATTTTTTTCGCCTGATT
**PB-42**	GGCGAACGATTTAGAGCTTGACGGTTGAATT
**PB-43**	ACCTTTTTATTTAACAATTTCATGGAAAGCC
**PB-44**	AGGGATTTAACAATATTACCGCCAGCCATTG
**PB-45**	CAACAGGAGAGCTAAACAGGAGGCCGATTAA

Supplementary Table 4 Modified staples for Antigen-PNA binding. Overhang sequences are underlined.

**Name**	**Sequence**
**PB-17 w/overhang**	GATTTTCAACCGCCTGCAACAGTGCCACGCTTTACTGGACTG
**PB-19 w/overhang**	ACGTGAACACAGTAACAGTACCTTTTACATCTTACTGGACTG
**PB-22 w/overhang**	ACGTGCTTTGGGGTCGAGGTGCCGTAAAGCATTACTGGACTG
**PB-25 w/overhang**	AAATGGATCGAACGAACCACCAGCAGAAGATTTACTGGACTG
**PB-27 w/overhang**	CAGTGAGGAATCTTGAGAAGTGTTGCCGCGCTTACTGGACTG

**PB-32 w/overhang**	CGCGCAGAGCTTTGAATACCAAGTAATTGAATTACTGGACTG
**PB-35 w/overhang**	ATATCAAAGCATCACCTTGCTGACGAACTGATTACTGGACTG
**PB-38 w/overhang**	AAACTATCACTTGCCTGAGTAGAAATAAAAGTTACTGGACTG
**PB-42 w/overhang**	GGCGAACGATTTAGAGCTTGACGGTTGAATTTTACTGGACTG
**PB-45 w/overhang**	CAACAGGAGAGCTAAACAGGAGGCCGATTAATTACTGGACTG

Supplementary Table 5 Modified staples with overhang to hybridize CpG sequences.

Overhang sequences are underlined.

**Name**	**Sequence**
**PB-5 w/overhang**	ATATAATCGTTGGCAAATCAACAGTAGAAAGTAAGTTAGGACCATGAAGT
**PB-6 w/overhang**	GAATTGAGTATCAGATGATGGCAATTCATCATAAGTTAGGACCATGAAGT
**PB-7 w/overhang**	CGCTGGCAAAGCGAAAGGAGCGGGTATTAATTAAGTTAGGACCATGAAGT
**PB-8 w/overhang**	TTTAAAAGCCTTTGCCCGAACGTCGCTAGGGTAAGTTAGGACCATGAAGT
**PB-9 w/overhang**	GCAATACTCCATCACGCAAATTAATAGACTTTAAGTTAGGACCATGAAGT
**PB-10 w/overhang**	TACAAACAAGGATTTAGAAGTATCCGTTGTATAAGTTAGGACCATGAAGT
**PB-12 w/overhang**	AGGAGCGGGCGGAACAAAGAAACATGATGAATAAGTTAGGACCATGAAGT
**PB-13 w/overhang**	ACAAACATACCTGAGCAAAAGAAGCACCAGATAAGTTAGGACCATGAAGT
**PB-14 w/overhang**	ATTAGAGCTAGGTGCACTAACAATAAGAATATAAGTTAGGACCATGAAGT
**PB-15 w/overhang**	CGTGGCACTTCTGACCTGAAAGCGCTAATAGTAAGTTAGGACCATGAAGT

Supplementary Table 6 Peak areas from mass spectra of each protein in Supplementary.

			**Peak Area**	
	Concentration of standards (ng/ml)	Internal Standard	Protein G	RBD
**1**	50	2.48E+05	2.02E+06	1.92E+06
**2**	40	2.06E+05	1.45E+06	1.42E+06
**3**	30	1.63E+05	1.22E+06	1.16E+06

**4**	20	1.05E+05	7.06E+05	7.35E+05
**5**	10	5.73E+04	3.75E+05	3.91E+05
**6**	5	4.15E+04	1.85E+05	1.99E+05
**Sample**		4.84E+05	4.22E+05	1.37E+06
		Normalized	8.73E+05	2.83E+06

Supplementary Table 7 FRET-dye modified staple strands.

**Name**	**Sequence**
**Fret1-Don1**	FAM//ATTCGACAACTTTTTCGTATTAAATTTTGAGTAACATTTTTTTATCATTTTAATTATCATCATTTTTATTCCTGAT
**Fret1-Acc1**	TAMRA//TACAAACAAGGATTTAGAAGTATCCGTTGTA
**Fret2-Don2**	FAM//TCCTCGTTGGATTTTTATCAGAGCGGAAAACGCTCATTTTTTGGAAATACCTGGTGAGGCGGTTTTTTCAGTATTAAC
**Fret2-Acc2**	TAMRA//ACGTGCTTTGGGGTCGAGGTGCCGTAAAGCA

Supplementary Table 8 Examples of Kd extracted from the literature for SARS-CoV-2 RBD binding to the human ACE2 based on BLI and SPR results.

**Receptor-Ligand**	**Kd (nM)**
**ACE2-RBD**	4.7 [1]
**ACE2-RBD**	3.2 [2]
**ACE2-RBD, Fc**	7.16 [2]
**ACE2-RBD dimer**	1.25 [2]
**ACE2-RBD**	1.59 [3]
**ACE2-RBD**	19.3[4]
**ACE2-RBD trimer**	0.04[4]

Supplementary Table 9 Clinical monitoring chart in animal study.

Appearance	Mobility	Attitude	Body Condition	TOTAL SCORE
0 - Smooth coat, bright eyes	0 - Active, exploring	0 - Alert	0 - Obese or Normal	0-5 = Normal daily monitoring
1 - Slightly scruffy and/or hunched at rest	1 - Walking, less active	1 - Mildly Lethargic	1 - Underconditioned	6-10 = 2x daily monitoring
2 - Scruffy and/or hunched at rest	2- Slow movement	2 - Lethargic	2 - Emaciated	≥11 = Euthanize
3 - Very scruffy and/or hunched, mild eye crust	3 - No movement	3 - Unaware		FD = Found Dead
4 - Very scruffy and/or hunched, closed eyes	4 - Unresponsive	Moist feed & feed on floor given post-infection		S or E = sacrificed/euthanized per study design

Supplementary Table 10 Scoring animals based on physical distress parameters (‘1’: PB NP; ‘2’: PB-CpG NP; ‘3’: 1 μg RBD-PB NP; ‘4’: 5 μg RBD-PB NP. X indicates the euthanized animals).

	Day	Time	Appearance					Mobility					Attitude					Body Condition					**TOTAL SCORE**				
**1**	Day 6	AM	1	2	1	1	1	1	1	1	1	1	1	1	0	1	1	0	0	0	0	0	**3**	**4**	**1**	**3**	**3**
**1**	Day 7	AM	4	4	1	4	4	3	3	1	3	3	3	3	0	3	3	2	2	0	2	2	**12**	**12**	**2**	**12**	**12**
**2**	Day 6	AM	1	1	1	1	0	1	1	1	0	0	1	1	1	0	0	0	0	0	0	0	**3**	**3**	**3**	**1**	**0**
**2**	Day 7	AM	4	4	4	1	1	3	3	3	1	1	2	2	2	0	0	2	2	2	0	0	**11**	**11**	**11**	**2**	**2**
**2**		PM																					**X**	**X**	**X**		
**2**	Day 8	AM				1					2	1				1					0		**X**	**X**	**X**	**4**	**1**
**2**	Day 9	AM				4					3					3					2		**X**	**X**	**X**	**12**	**0**
**2**		PM																								**X**	
**3**	Day 6	AM	1	1	2	1	0	2	2	2	2	0	1	1	1	1	1	0	0	0	0	0	**4**	**4**	**5**	**4**	**1**
**3**	Day 7	AM	4	4	4	4	1	4	4	4	4	1	3	3	3	3	1	1	1	1	1	1	**12**	**12**	**12**	**12**	**4**

**3**		PM																					**X**	**X**	**X**	**X**	
**3**	Day 8	AM					4					4					3					1	**X**	**X**	**X**	**X**	**12**
**3**		PM																									**X**
**4**	Day 8	AM	0	0	4	4	0	0	1	4	4	1	0	0	3	3	0	0	0	1	1	0	**0**	**1**	**12**	**12**	**1**

SUPPLEMENTARY FIGURES

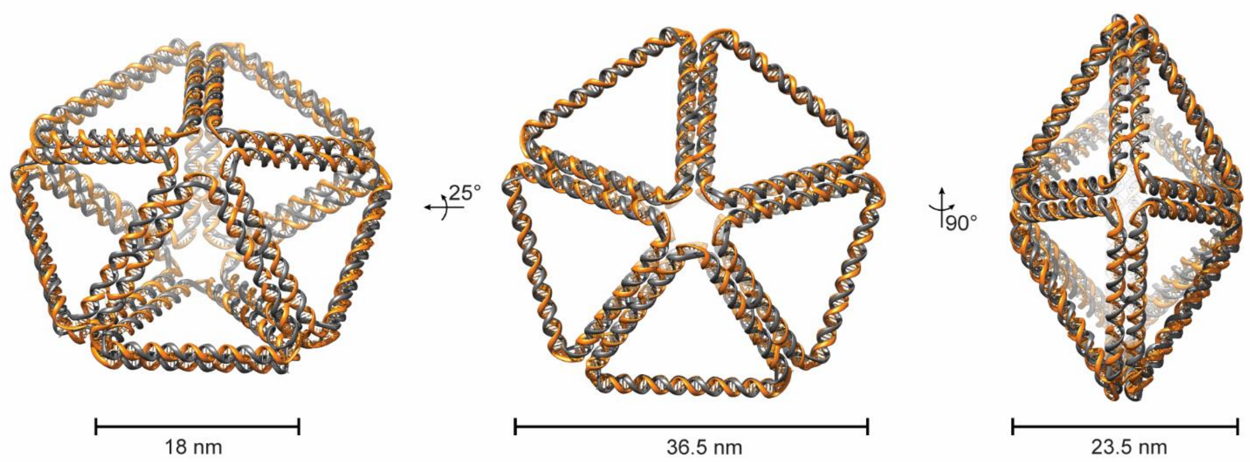

Supplementary Figure 1 Design of DNA pentagonal bipyramid (PB). PB was designed with DAEDALUS and the rendering performed with Chimera.

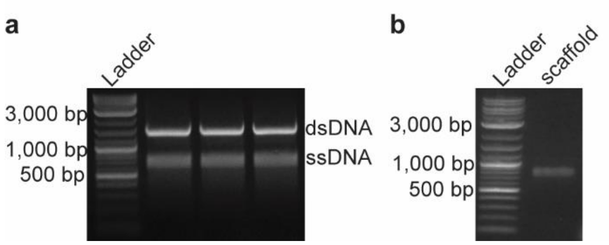

Supplementary Figure 2 PB ssDNA scaffold production. The 1,616 nts ssDNA scaffold was synthetized with aPCR. **a** Agarose gel electrophoresis showing the representative result of n=3 independent aPCR reactions. **b** Validation of the ssDNA scaffold purity.

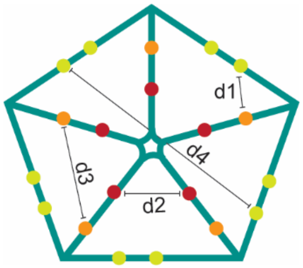

Supplementary Figure 3 Graphical representation of the distances between the antigen-binding sites on the PB NP. (d: distance) d1= 9 nm; d2= 11 nm; d3= 15 nm; d4= 36.5 nm. Distances were measured in Chimera using the model generated by DAEDALUS. Each color depict the overhangs at specific locations to define the closest and farthest distances.

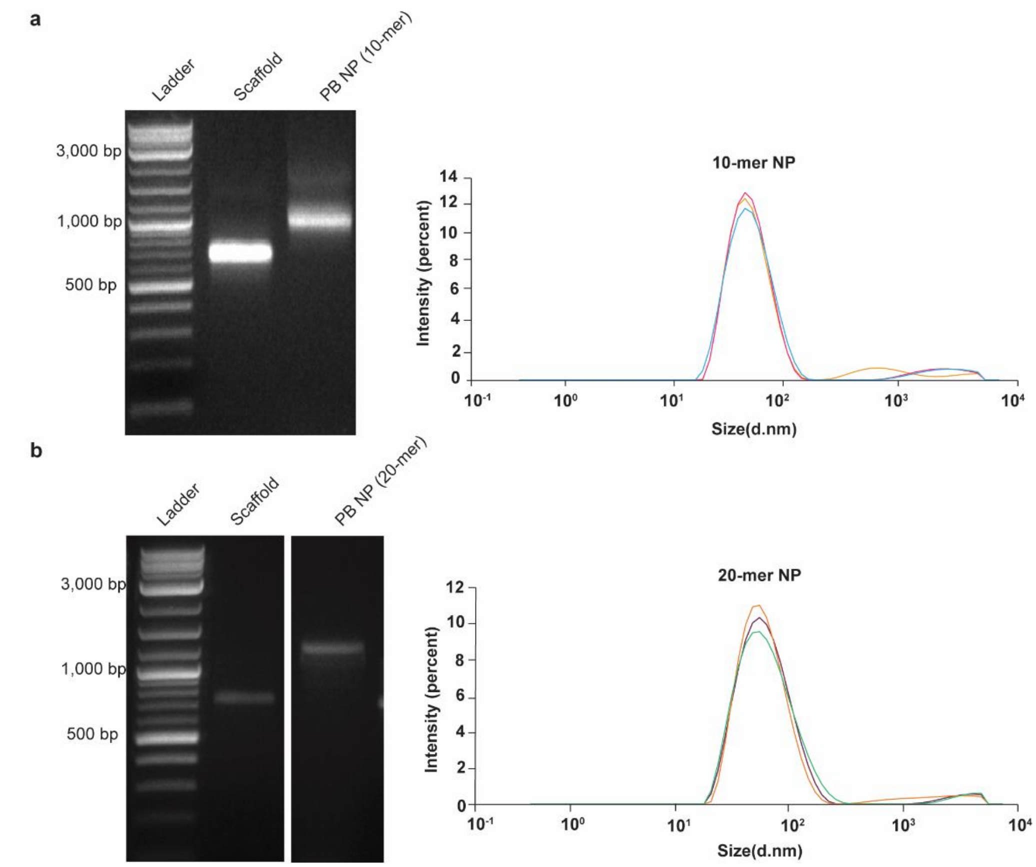

Supplementary Figure 4 Assessment of folding PB nanoparticles. a Characterization of PB-10 (10 overhangs on one face of PB) via agarose gel electrophoresis and DLS. Hydrodynamic diameter (Z-average= 47.6 nm) measured from n=3 technical replicates of NP preparations. **b** Characterization of PB-20 (20 overhangs on each face of PB) via agarose gel electrophoresis and DLS. Hydrodynamic diameter (Z-average= 51.2 nm) measured from n=3 technical replicates of NP preparations. Theoretical diameters were estimated at 40.2 nm and 46.4 nm for PB-10 and PB-20, respectively. Polydispersity index measured for PB-10 has an average of 0.241 and for PB-20 is 0.238.

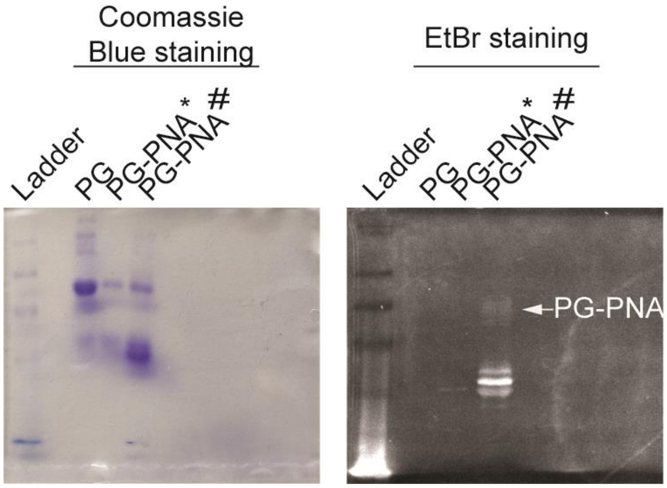

Supplementary Figure 5 Validation of PNA-maleimide conjugated PG in denaturing gel. 10% SDS-PAGE (sodium dodecyl sulfate polyacrylamide gel electrophoresis) gel was initially stained with PageBlue protein staining solution to visualize PG. Then the same gel was incubated with EtBr to visualize PNA-conjugated PG. White arrow indicates PG-PNA. Approximate molecular mass of PG is 21.9 kDA and migrate in SDS-PAGE with molecular weight of 40 kDA. Theoretical mass of PG-PNA is 24.9 kDA. * indicate that the PG was reacted with PNA without TCEP reduction, # indicate the PG-PNA was first reduced with TCEP and then reacted with PNA overnight.

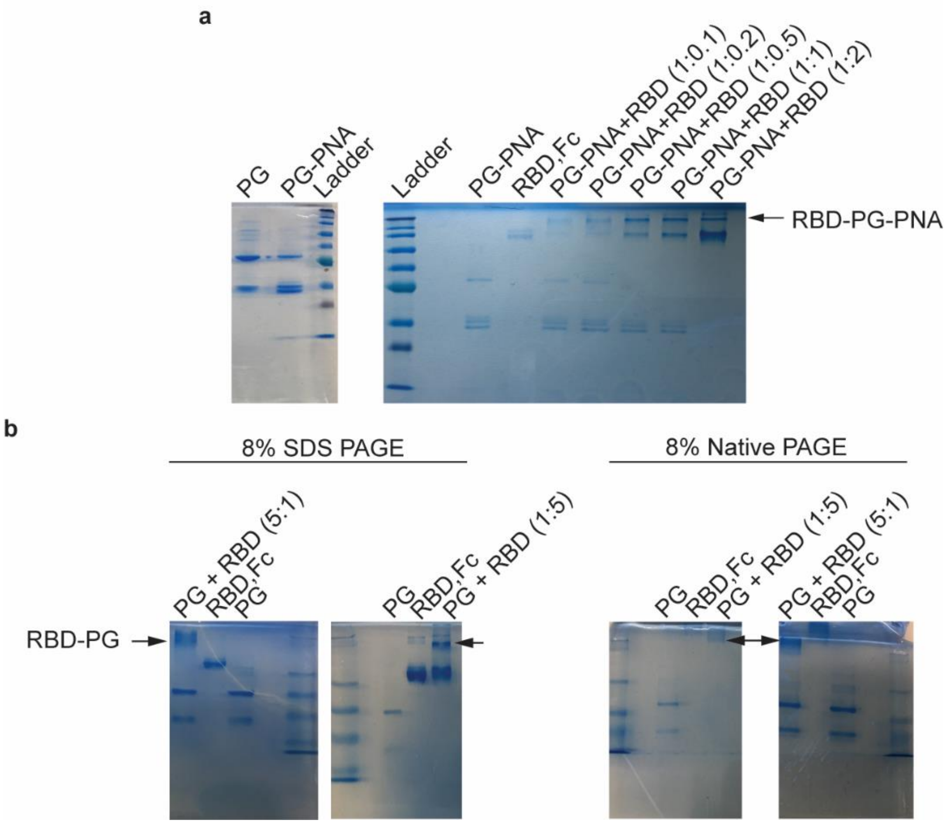

Supplementary Figure 6 Formation of the RBD trimer. a PG-PNA is incubated with different molar ratios of RBD-Fc. The conjugation of two proteins was evaluated through running the samples in SDS-PAGE gel. **b** Additionally, five-fold excess of PG without PNA was incubated with RBD-Fc, and five-fold excess of RBD-Fc was incubated with PG without PNA. Conjugation of two proteins was examined via native and denaturing gel electrophoresis. Theoretical molecular mass of RBD, Fc is 51.5 kDA and migrate in SDS PAGE approximately between 60 and 65 kDA. Theoretical mass of RBD-PG is 176.4 kDA and RBD-PG-PNA is 179.4 kDA.

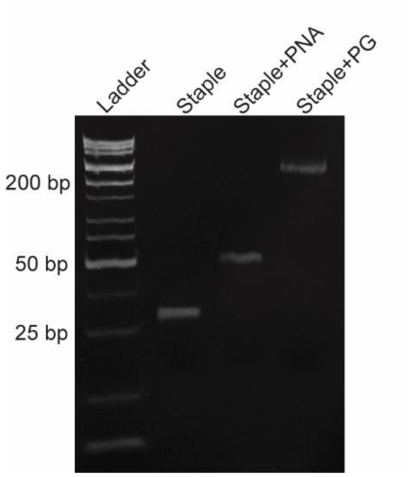

Supplementary Figure 7 Protein G-PNA binding to the DNA overhangs. An individual staple strand with an overhang was incubated with PNA and PG-PNA at 37°C. We used 14% PAGE gel to evaluate the hybridization of PNA alone and PG-PNA to the overhang.

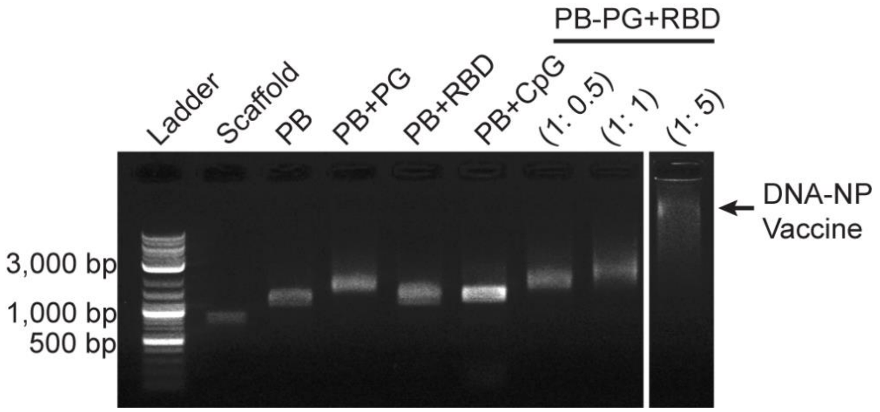

Supplementary Figure 8 Agarose gel electrophoresis for validation of PG-RBD complex attachment to DNA-NP, along with CpG hybridization.

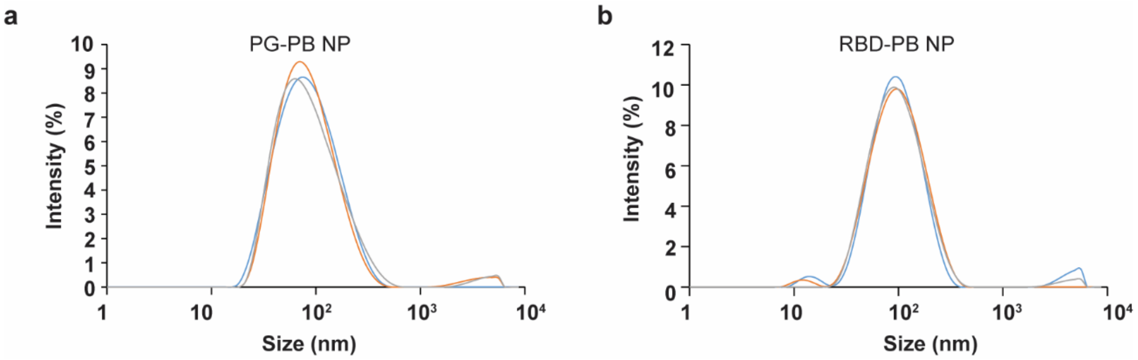

Supplementary Figure 9 Dynamic light scattering results providing comparison of the hydrodynamic diameter after each step of nanoparticle conjugation (n=3 technical replicates).

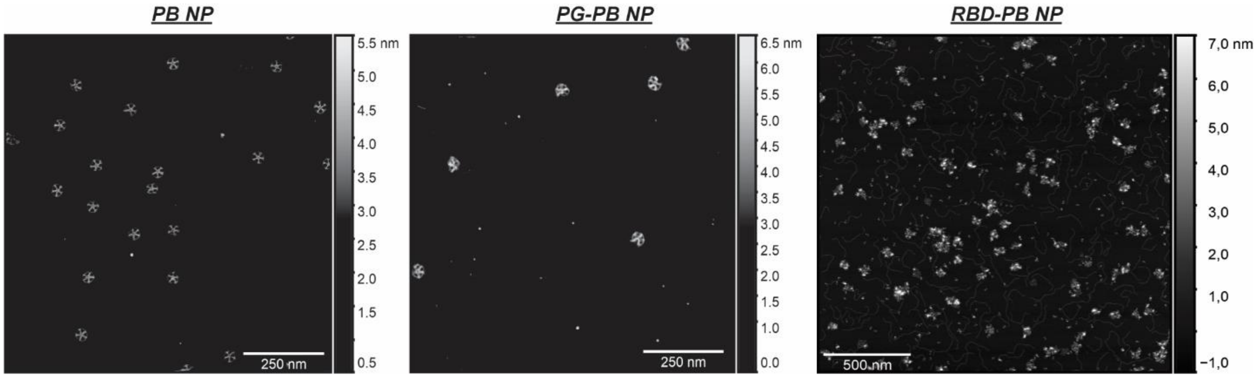

Supplementary Figure 10 Representative full AFM images of PB nanoparticles, PG-modified PB nanoparticles, and RBD-attached PB nanoparticles.

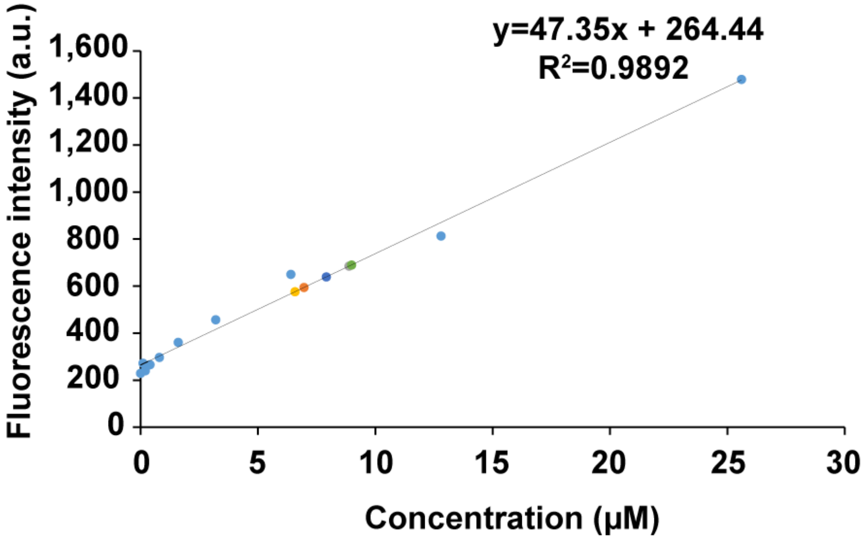

Supplementary Figure 11 Representative tryptophan emission standard curve for PB NP coverage by PG. Orange dot: PG-PB DNA-NP (1-mer), grey dot: PG-PB DNA-NP (2-mer), yellow dot: PG-PB DNA-NP (5-mer), dark blue dot: PG-PB DNA-NP (10-mer), green dot: PG-PNA.

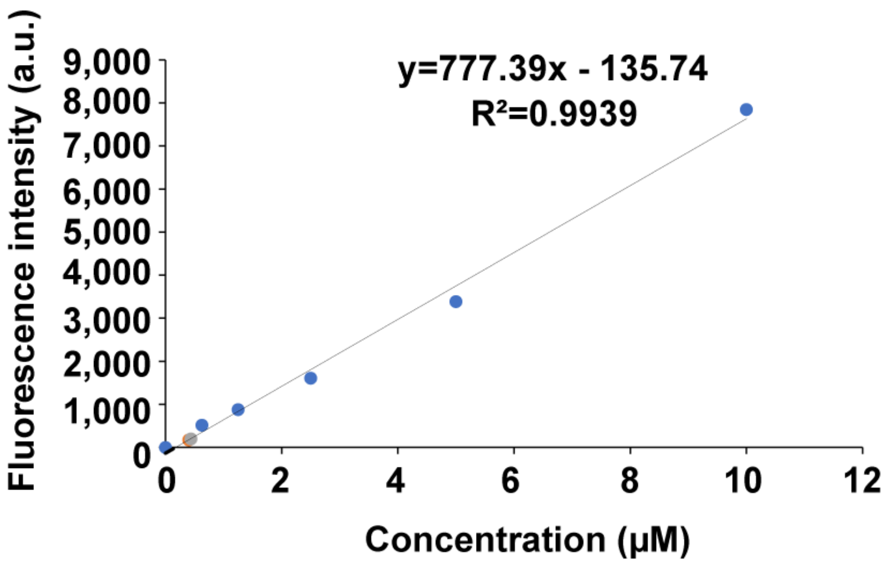

Supplementary Figure 12 Representative Cy5 emission standard curve for PB NP coverage by RBD. Orange dot depicts RBD-Cy5 and grey dot is for Cy5-RBD-PB DNA-NP, which have same concentration of RBD-Cy5 to show coverage of PB DNA-NP by RBD.

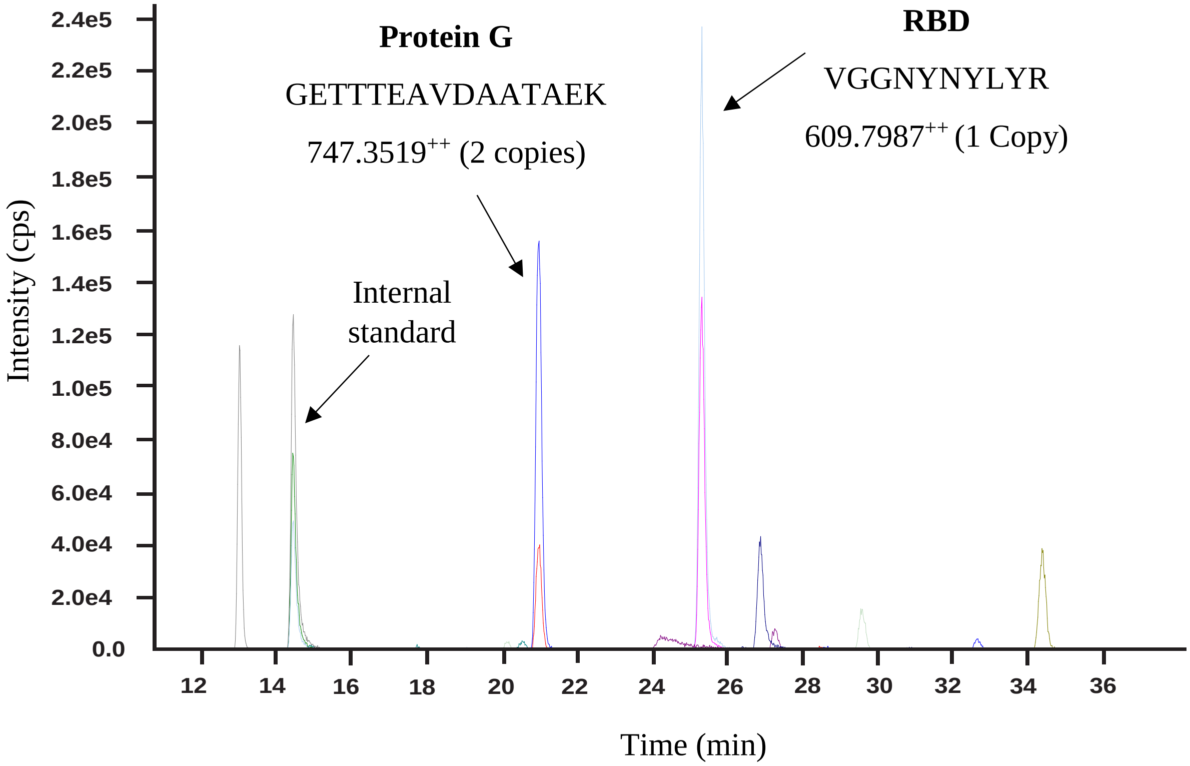

Supplementary Figure 13 Mass spectra based on the m/z transitions calculated for Protein G and RBD.

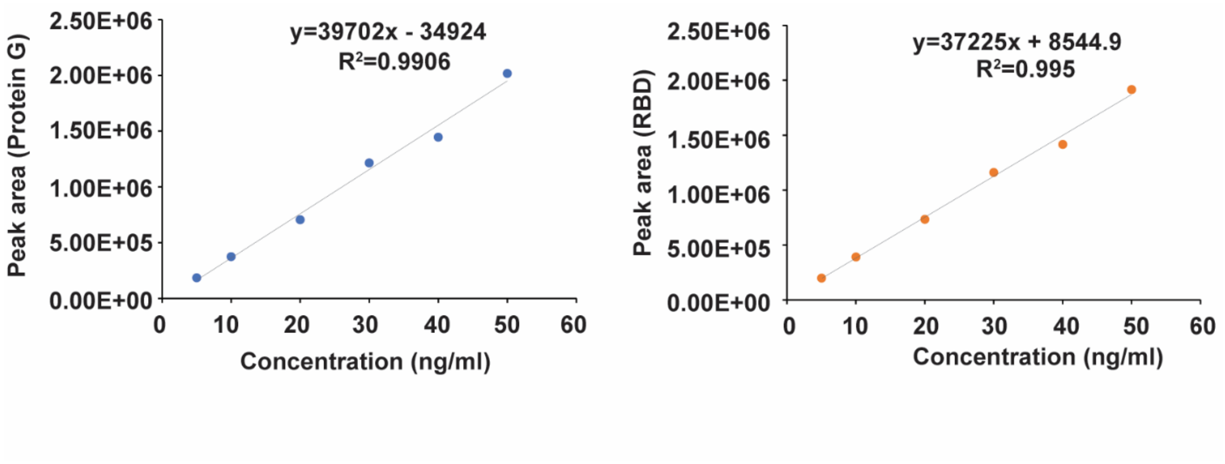

Supplementary Figure 14 Standard curves established through correlation from the peak areas of each protein and concentration of standards .

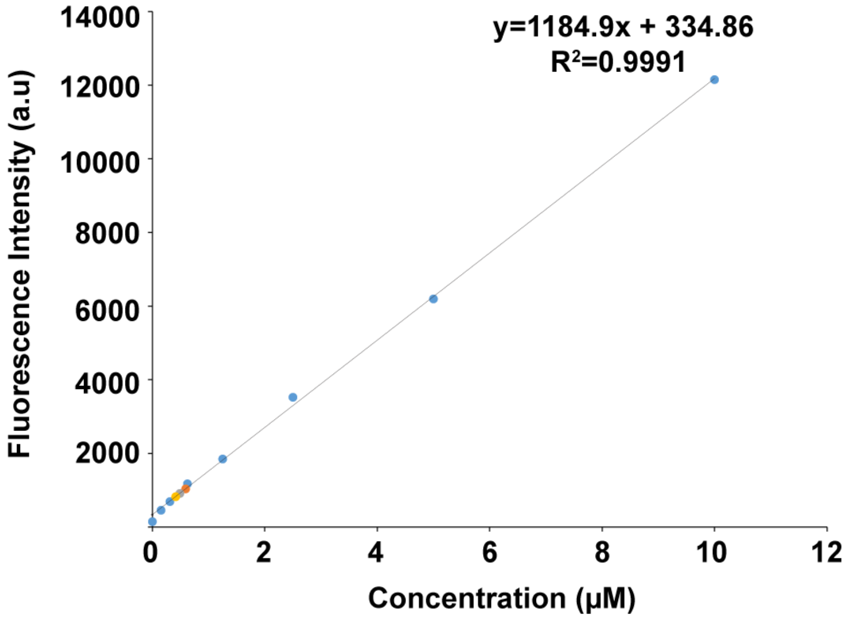

Supplementary Figure 15 Quantification of CpG coverage on DNA-NP. The graph displays a representative fluorescein-labelled CpG ODN standard curve with three experimental point of the DNA-NPs with 10 overhangs for CpG conjugation. Orange, grey, and yellow dots refers to three separate DNA NP-CpG preparations at the same concentrations.As a results of n=3 independent samples, we determine the CpG coverage to be 79.7% ± 14.3.

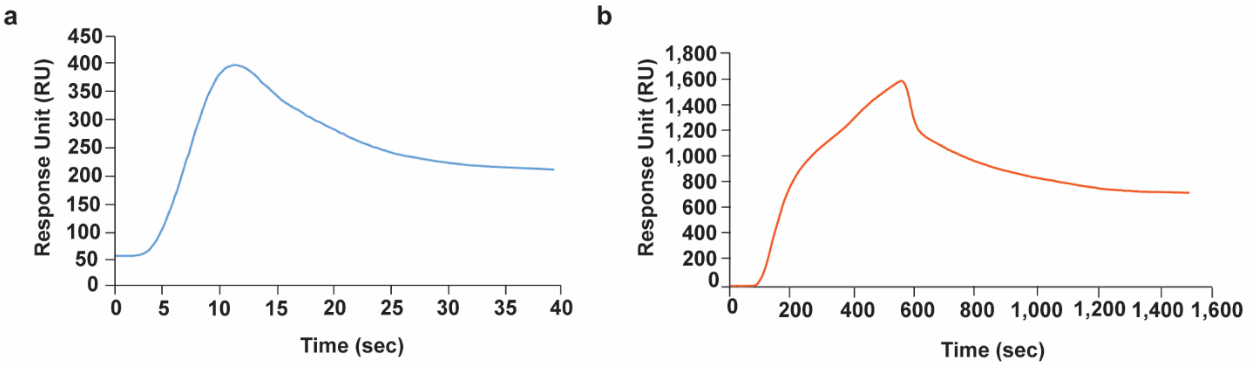

Supplementary Figure 16 Representative SPR binding curve. **a** Streptavidin binding on biotinylated gold sensor. **b** Immobilization of the ACE2-biotinylated receptor on streptavidin-modified gold sensor.

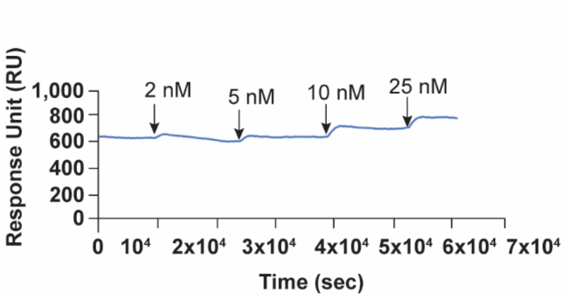

Supplementary Figure 17 Sequential binding interactions of RBD with the ACE2 receptor for four different concentrations of RBD-PB NP during single cycle kinetics (SCK) experiment.

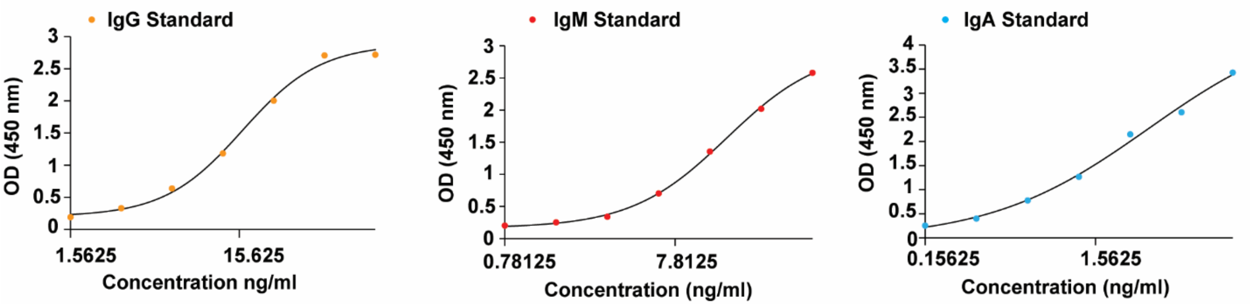

Supplementary Figure 18 ELISA standard curves prepared for the assessment of serum IgG, IgM, and IgA concentration (ng/ml).

SUPPLEMENTARY REFERENCES

1. Lan, J. *et al.* Structure of the SARS-CoV-2 spike receptor-binding domain bound to the ACE2 receptor. *Nature* **581**, 215–220 (2020).

2. Pan, X. *et al.* RBD-homodimer, a COVID-19 subunit vaccine candidate, elicits immunogenicity and protection in rodents and nonhuman primates. *Cell Discov* **7**, 82 (2021).

3. Zhang, Z. *et al.* Potent prophylactic and therapeutic efficacy of recombinant human ACE2-Fc against SARS-CoV-2 infection in vivo. *Cell Discov* **7**, 65 (2021).

4. Routhu, N. K. *et al.* SARS-CoV-2 RBD trimer protein adjuvanted with Alum-3M-052 protects from SARS-CoV-2 infection and immune pathology in the lung. *Nat Commun* **12**, 3587 (2021).
