# Supplementary material for: DNA origami presenting the receptor binding domain of SARS-CoV-2 elicit robust protective immune response
Source: Commun Biol. 2023 Mar 23;6:308. doi: 10.1038/s42003-023-04689-2 (PMC10034259; doi:10.1038/s42003-023-04689-2)
Supplement: Supplementary file 3 — Description of Additional Supplementary Files [file 42003_2023_4689_MOESM3_ESM.pdf]

Description of Additional Supplementary Files

File name: Supplementary Data 1
Description: This Excel file contains the raw data used to prepare the graphs in the different figures displayed in the main text and the supplementary information document.
