# Supplementary material for: DNA origami presenting the receptor binding domain of SARS-CoV-2 elicit robust protective immune response
Source: Commun Biol. 2023 Mar 23;6:308. doi: 10.1038/s42003-023-04689-2 (PMC10034259; doi:10.1038/s42003-023-04689-2)
Supplement: Supplementary file 5 — Reporting Summary [file 42003_2023_4689_MOESM5_ESM.pdf]

nature portfolio

Corresponding author(s): Remi Veneziano

Last updated by author(s): Feb 28, 2023

Reporting Summary

Nature Portfolio wishes to improve the reproducibility of the work that we publish. This form provides structure for consistency and transparency in reporting. For further information on Nature Portfolio policies, see our Editorial Policies and the Editorial Policy Checklist.

Statistics

For all statistical analyses, confirm that the following items are present in the figure legend, table legend, main text, or Methods section.

n/a	Confirmed	
☐	☒	The exact sample size (n) for each experimental group/condition, given as a discrete number and unit of measurement
☐	☒	A statement on whether measurements were taken from distinct samples or whether the same sample was measured repeatedly
☐	☒	The statistical test(s) used AND whether they are one- or two-sided<br>*Only common tests should be described solely by name; describe more complex techniques in the Methods section.*
☒	☐	A description of all covariates tested
☒	☐	A description of any assumptions or corrections, such as tests of normality and adjustment for multiple comparisons
☐	☒	A full description of the statistical parameters including central tendency (e.g. means) or other basic estimates (e.g. regression coefficient) AND variation (e.g. standard deviation) or associated estimates of uncertainty (e.g. confidence intervals)
☐	☒	For null hypothesis testing, the test statistic (e.g. F, t, r) with confidence intervals, effect sizes, degrees of freedom and P value noted<br>*Give P values as exact values whenever suitable.*
☒	☐	For Bayesian analysis, information on the choice of priors and Markov chain Monte Carlo settings
☒	☐	For hierarchical and complex designs, identification of the appropriate level for tests and full reporting of outcomes
☒	☐	Estimates of effect sizes (e.g. Cohen's d, Pearson's r), indicating how they were calculated

Our web collection on statistics for biologists contains articles on many of the points above.

Software and code

Policy information about availability of computer code

Data collection	Surface Plasmon Resonance data collection was done with the Nicoya OpenSPR Instrument using the Open SPR software version 4.3.7704.38211. The gel images were acquired on an Azure c150 imager with the software version 2.1.3.0710. Fluorescence measurements were done on a fluorescent plate reader Tecan Safire2 with the Magellan software version 6.6. The Dynamic light scattering measurements were done on a Malvern Nanozetasizer using the Malvern Zetasizer Software version 7.13. Mass spectrometry data were acquired with a Sciex QTRAP 4500 mass spectrometer equipped with a Shimadzu Prominence UFLC XR System acquired using the the Analyst software 1.7. AFM data were collected on a JPK instruments NanoWizard 4 fast-scan AFM with the software JPK NanoWizard Control Software v6, version 6.1.198.
Data analysis	The PRNT assay analysis was done with GraphPad prism version 9.0.2. Statistical analysis were done with R studio version 1.2.5033 and JASP version 0.16.4.0. Surface plasmon resonance data analysis were done with the TraceDrawer software version 1.9.2. Microsoft office Excel 2016 was used for plotting the FRET assays results and the survival experiments. The AFM images were processed with Gwyddion (version 2.62) and analyzed with GIMP version 2.10.28. Mass spectrometry data were analyzed via Analyst software 1.7.

For manuscripts utilizing custom algorithms or software that are central to the research but not yet described in published literature, software must be made available to editors and reviewers. We strongly encourage code deposition in a community repository (e.g. GitHub). See the Nature Portfolio guidelines for submitting code & software for further information.

Data

Policy information about availability of data

All manuscripts must include a data availability statement. This statement should provide the following information, where applicable:

- Accession codes, unique identifiers, or web links for publicly available datasets
- A description of any restrictions on data availability
- For clinical datasets or third party data, please ensure that the statement adheres to our policy

All raw and processed data can be made available upon reasonable request to the corresponding authors.

Field-specific reporting

Please select the one below that is the best fit for your research. If you are not sure, read the appropriate sections before making your selection.

☒ Life sciences ☐ Behavioural & social sciences ☐ Ecological, evolutionary & environmental sciences

For a reference copy of the document with all sections, see nature.com/documents/nr-reporting-summary-flat.pdf

Life sciences study design

All studies must disclose on these points even when the disclosure is negative.

Sample size	Gel electrophoresis presented are representative results but each gel has been done at least 3 times each with distinct replicates. For SPR experiments all measurements were done with n> or = to 3. FRET stability studies of the DNA nanoparticles were done with the following sample size: n=3 for Bare nanoparticles in serum (negative control); n=3 for CpG-PB nanoparticles; n=3 for RBD-CpG-PB nanoparticles; n=3 for RBD-PB nanoparticles. For the immunization study, we prepared five different control groups and four different vaccine constructs. 5 animals were used for each of the 4 different vaccine constructs and 5 different control groups. For the viral challenge study, we used five different vaccine constructs (PB-CpG was included)(N=5 mice per sample) and control groups (N=5 mice per sample). For the antibody durability assessment, one vaccine construct (N=5 mice per sample) and one placebo sample (N=5 mice per sample) were used. ELISA analysis were performed for the serum samples collected from the animals (N=4 serum sample) injected with one vaccine construct and placebo.
Data exclusions	No data were excluded.
Replication	In vitro experiments were performed multiple times on different days with distinct nanoparticle preparations to confirm our observations. For the animal experiments, multiple animals were used to allow statistical analysis of the results obtained. The immunization study and the viral challenge assay were done with different nanoparticles preparations. All nanoparticles preparation were carefully characterized to ensure reproducibility.
Randomization	Not relevant to this study.
Blinding	The investigators were not blind to the nanoparticles used, but the animal experiments have been performed by different investigators than the ones that prepared the nanoparticles and run the in vitro experiments.

Reporting for specific materials, systems and methods

We require information from authors about some types of materials, experimental systems and methods used in many studies. Here, indicate whether each material, system or method listed is relevant to your study. If you are not sure if a list item applies to your research, read the appropriate section before selecting a response.

Materials & experimental systems

n/a	Involved in the study	
☒	☐	Antibodies
☐	☒	Eukaryotic cell lines
☒	☐	Palaeontology and archaeology
☐	☒	Animals and other organisms
☒	☐	Human research participants
☒	☐	Clinical data
☒	☐	Dual use research of concern

Methods

n/a	Involved in the study	
☒	☐	ChIP-seq
☒	☐	Flow cytometry
☒	☐	MRI-based neuroimaging

Eukaryotic cell lines

Policy information about cell lines

Cell line source(s)	Vero Cells from ATCC (CCL-81)
Authentication	The cells were provided by a reliable source (ATCC) that performed quality control upon shipment. We do not performed specific authentication. Upon receipt of the cell vials, they are expanded and multiple vials are frozen. We normally thaw out a new vial of cells once the passage number gets too high.
Mycoplasma contamination	The cells are provided by ATCC as mycoplasma free. We did not perform any extra control for mycoplasma contamination but we did check that the cells were healthy under microscope before any of our studies.
Commonly misidentified lines (See ICLAC register)	N/A

Animals and other organisms

Policy information about studies involving animals; ARRIVE guidelines recommended for reporting animal research

Laboratory animals	6 to 8 weeks-old male K18-Ace2 (B6.Cg-Tg(K18-ACE2)2Prlmn/J) mice were used for the viral challenge assay and 6 to 8 weeks old female BALB/c mice were used for the immunization assay
Wild animals	That study does not involve wild animals.
Field-collected samples	That study does not involve field-collected samples.
Ethics oversight	All animal studies carried out for these studies were in accordance with recommendations of the Institutional Animal Care and Use Committee (IACUC protocol #0399) at George Mason University.

Note that full information on the approval of the study protocol must also be provided in the manuscript.
